# Fuzzy-FishNET: a highly reproducible protein complex-based approach for feature selection in comparative proteomics

**DOI:** 10.1186/s12920-016-0228-z

**Published:** 2016-12-05

**Authors:** Wilson Wen Bin Goh

**Affiliations:** School of Pharmaceutical Science and Technology, Tianjin University, Tianjin, People’s Republic of China

**Keywords:** Proteomics, Networks, Bioinformatics, GSEA, QPSP, SNET, FSNET, PFSNET, Renal Cancer

## Abstract

**Background:**

The hypergeometric enrichment analysis approach typically fares poorly in feature-selection stability due to its upstream reliance on the *t*-test to generate differential protein lists before testing for enrichment on a protein complex, subnetwork or gene group.

**Methods:**

Swapping the *t*-test in favour of a fuzzy rank-based weight system similar to that used in network-based methods like Quantitative Proteomics Signature Profiling (QPSP), Fuzzy SubNets (FSNET) and paired FSNET (PFSNET) produces dramatic improvements.

**Results:**

This approach, Fuzzy-FishNET, exhibits high precision-recall over three sets of simulated data (with simulated protein complexes) while excelling in feature-selection reproducibility on real data (based on evaluation with real protein complexes). Overlap comparisons with PFSNET shows Fuzzy-FishNET selects the most significant complexes, which are also strongly class-discriminative. Cross-validation further demonstrates Fuzzy-FishNET selects class-relevant protein complexes.

**Conclusions:**

Based on evaluation with simulated and real datasets, Fuzzy-FishNET is a significant upgrade of the traditional hypergeometric enrichment approach and a powerful new entrant amongst comparative proteomics analysis methods.

**Electronic supplementary material:**

The online version of this article (doi:10.1186/s12920-016-0228-z) contains supplementary material, which is available to authorized users.

## Background

Mass spectrometry (MS)-based proteomics is becoming increasingly important in contemporary biological and clinical research [1]. Yet, despite significant technological advancement marking quantum leaps in protein extraction and spectra-acquisition [2–4], data reliability issues in MS-based proteomics persist: the primary issues being incomplete proteome coverage and inter-sample protein identification inconsistency [5]. These problems are not yet resolved satisfactorily on current proteomics paradigms [6–8]. Moreover, with the advent of brute-force spectra capture strategies e.g. Data-Independent Acquisition (DIA) [9, 10], increased noise becomes an inadvertent consequence, and contribute yet another layer of complexity [2].

Proteomics allows the simultaneous expressional profiling of thousands of proteins (although leaving thousands more which remain undetected). The first order of business is usually to identify proteins which are strongly and consistently differential, with the expectation that these are phenotypically relevant. This process is known as “feature selection”, and helps to concentrate analysis on a smaller feature (protein) set which is easier to study, understand and validate experimentally [11]. Unlike animal models or cell lines, clinical samples are highly heterogeneous, reflecting different disease etiologies and genetic backgrounds amongst unique individuals [12]. Heterogeneity, compounded with the fact that different proteins being identified between samples [13], and possible quantification accuracy issues [14] means that in practical deployment, it is difficult to make reliable identification of useful biomarkers or drug targets during analysis of clinical data. Hence, more sophisticated and robust analytical methods are required.

Contextualization at the level of subnets, or more specifically, protein complexes, can resolve proteomic coverage and consistency issues [15–18]. Use of protein complexes as features for feature-selection instead of predicted clusters from reference networks, is a more powerful approach as protein complexes are enriched for biological signal [19]. However, use of protein complexes alone (despite its high biological signal enrichment) is insufficient: the nature of the statistical analysis method is also equally important. The hypergeometric enrichment (HE) test is commonly used in many areas of biological research from testing for functional enrichment [20–25] to testing for over-representation of genes in predicted subnetworks [26]. Yet, despite its wide use, even when used with protein complexes, HE does poorly, particularly in terms of feature-selection stability [16].

HE is actually a two-part test (see Methods). But its reliance on the *t*-test to generate a differential protein list for subsequent enrichment analysis based on the hypergeometric test is a known contributing factor towards its high instability [27, 28], and is demonstrated again in recent work [16]. We may redesign HE using elements of design that have worked well in other techniques.

QPSP, and the rank-based network approaches (RBNAs), SNET (SubNET) [29], FSNET (Fuzzy SNET) and PFSNET (Paired FSNET) [30] have been shown to be highly stable and robust, these techniques are similar in that they use a fuzzy weighting system on proteins ranked by expression [31] (see Methods).

By incorporating the fuzzy weighting system into HE, and doing away with upstream *t*-test differential protein pre-selection, a new spin on the original HE technique, Fuzzy-FishNET, is introduced here. Its name comes from the incorporation of the fuzzy weighting system in QPSP/FSNET/PFSNET with the one-sided Fisher’s exact test (equivalent to the hypergeometric test). Fuzzy-FishNET is evaluated based on precision and recall on three simulated datasets, and also its stability/reproducibility on real data.

## Methods

### Simulated proteomics datasets --- D.1.2, D2.2 and RC1

Two simulated proteomics datasets, D1.2 and D2.2, from the study of Langley and Mayr are used [32]. D.1.2 is obtained from a study of proteomic changes resulting from addition of exogenous matrix metallopeptidase (3 control, 3 test) while D2.2 is obtained from a study of hibernating arctic squirrels (4 control, 4 test). Protein quantification in both studies is based on spectral counts.

For both D1.2 and D2.2, 100 simulated datasets each with 20% randomly generated differential features are generated. The 20% threshold is arbitrary, for D1.2 and D2.2, this corresponds to 177 and 710 differential proteins respectively. For a given feature measured amongst samples derived from two different sample classes A and B, the effect size is the magnitude of the inter-class difference e.g. the differences of the means amongst samples derived from classes A and B. Here, the effect sizes of these 20% differential features are randomly selected from one out of five possibilities or p (20%, 50%, 80%, 100% and 200%), increased in one class and not in the other, and expressed as:

$$ S{C}_{i,j}\hbox{'}=S{C}_{i,j}\ *\ \left(1+\mathrm{p}\right) $$

where SC_i,j_ and SC_i,j_’ are respectively the original and simulated spectral count from the j^th^ sample of protein i.

RC1 comes from the 12 controls from the renal cancer (RC) dataset (see below). As with D1.2 and D2.2, 20% random proteins are randomly selected as differential, an effect size sampled from one of 5 possibilities, and inserted in only half of the controls, thus creating 6 control and 6 artificial test samples. This is also repeated 100 times to generate 100 simulated datasets.

### Proteomics dataset --- renal cancer (RC)

The renal cancer (RC) study of Guo et al. [2] is derived from six pairs of non-tumorous and tumorous clear-cell renal carcinoma (ccRCC) tissues based on the SWATH spectra-acquisition method. The six sample pairs are examined twice, as two different technical batches.

All SWATH spectra maps are analyzed using OpenSWATH [9] against a spectral library containing 49,959 reference spectra for 41,542 proteotypic peptides from 4,624 reviewed SwissProt proteins [2]. The library is compiled via library search of spectra captured in DDA mode (linking spectra mz and rt coordinates to a library peptide). Proteins are quantified via spectral count.

### Protein complexes (subnets)

Although subnets or clusters are predictable from large biological networks, real biological complexes are enriched for biological signal, far outperforming predicted complexes/subnets from reference networks [19, 31, 33, 34]. Here, known human protein complexes derived from the CORUM database are used [35].

To avoid high fluctuation in the test statistics used by some of the methods considered here (e.g. QPSP), complexes with at least 3 proteins that were identified and measured in the proteomics screen are retained (1363 complexes)

### Hypergeometric-enrichment (HE)

HE is a frequently used form of protein complex/subnetwork evaluation and consists of two steps [5]: First, differential proteins are identified using the two-sample *t*-test [36]. This is followed by a hypergeometric test where given a total of *N* proteins (with *B* of these belonging to a complex) and *n* test-set proteins (i.e., differential), the exact probability *P* that *b* or more proteins from the test set are associated by chance with the complex is given by [37]:

$$ P\left(X\ \ge b\right)={\displaystyle \sum_{i=b}^{\min \left(n,B\right)}}\frac{\left(\begin{array}{c}\hfill n\hfill \\ {}\hfill i\hfill \end{array}\right)\left(\begin{array}{c}\hfill N-n\hfill \\ {}\hfill B-i\hfill \end{array}\right)}{\left(\begin{array}{c}\hfill N\hfill \\ {}\hfill B\hfill \end{array}\right)} $$

The sum *P*(*X* ≥ *b*) is the *p*-value of the hypergeometric test.

### Gene-set enrichment analysis (GSEA)

The direct-group (DG) analysis approach, Gene-Set Enrichment Analysis, or GSEA is developed as a more powerful alternative to HE, as it obviates the *t*-test-based protein pre-selection step. In GSEA, a complex is tested by comparing the distribution of constituent protein expression between phenotype classes against that of proteins outside the complex using a Kolmogorov-Smirnov (KS) statistic [38].

Denoting proteins in the complex as the set D and proteins outside the complex as the set D’, the KS-statistic KS_D,D‘_is expressed as:

$$ K{S}_{D,D*}=ma{x}_x\left|{F}_{1,D}(x)-{F}_{2,D\hbox{'}}(x)\right| $$

where *F*_1,*D*_(*x*) and *F*_2,*D* *_(*x*) are respectively the number of proteins in D and D’ that whose rank is higher than the rank x. The null hypothesis is rejected at an alpha of 0.05 if

$$ K{S}_{D,D\hbox{'}}\ge c(alpha)*\sqrt{\frac{\left|D\left|+\right|{D}^{\hbox{'}}\right|}{\left|D\right|\ *\left|{D}^{\hbox{'}}\right|\ }} $$

where *c(alpha)* is the critical value at a given alpha level. Here, at an alpha of 0.05, *c(alpha)* = 1.36.

### Quantitative proteomics signature profiling (QPSP)

In QPSP, each sample is sorted based on abundance. The most abundant proteins above a certain percentile (the value is denoted as alpha1) are first selected. A second percentile value (defined as alpha2) is then used to extend the protein list [30]. To penalize lower-ranked proteins below alpha1 and above alpha2, proteins are assigned interpolated weights based on their ranks.

Alpha1 and alpha 2 are typically set as the top 10%, and top 20% ranks respectively. Rank-based weighting is achieved via discretization of the ranks from top 10-20% into four bins: 10–12.5% (weight 0.8), 12.5% to 15% (0.6), 15–17.5% (0.4), 17.5 to 20% (0.2). All other proteins beyond alpha2 (viz. remaining proteins) have a weight of 0 (and are thus ignored). Proteins above alpha1 are assigned a full weight of 1.

Given each sample, a vector of hit-rates is generated by considering the overlaps of the proteins (given their weights) against a vector of complexes. Given a sample in class A (S_A_) and a vector of complexes of length n, for each complex C_i_ in the complex vector, the hit-rate is the intersection of proteins in S_A_ and C_i_, modulated by the weight, over the total number of proteins in C_i_.

Let the hit-rate for S_A_ in C_i_ be H(S_A,_ C_i_). Therefore, the vector of hit-rates for sample S_A_ is H_SA_ = 〈H(S_A,_ C_1_)…, H(S_A,_ C_n_)〉. This vector of hit-rates signifies the sample’s signature profile based on complexes.

QPSP works with simple two-sample *t*-test. For each complex C in the complex vector, two lists are compared against each other, HA = 〈H(A_1_,C),…, H(A_m_,C)〉 and HB = 〈H(B_1_,C),…, H(B_n_,C)〉, where A and B are phenotype classes of lengths m and n respectively. The t-statistic, t_score, between HA and HB is computed by:

$$ t\_ score=\frac{\overline{HA}-\overline{HB}}{S_{HA,HB}\sqrt{\frac{1}{n}+\frac{1}{m}}} $$

where

$$ {S}_{HA,HB}=\sqrt{\frac{\left(m-1\right){S_{HA}}^2-\left(n-1\right){S_{HB}}^2}{m+n-2}} $$

If t_score for C_i_ is significant (i.e. its associated *p*-value falls below 0.05), then C_i_ is considered differential.

### SNET/FSNET/PFSNET

In SNET, given a protein *g*_*i*_ and a tissue *p*_*k*_, let *fs*(*g*_*i*_,*p*_*k*_) = 1, if the protein *g*_*i*_ is among the top alpha percent (default = 10%) most-abundant proteins in the tissue *p*_*k*_; and = 0 otherwise.

Given a protein *g*_*i*_ and a class of tissues *C*_*j*_, let

$$ \beta \left({g}_i,{C}_j\right)={\displaystyle \sum_{p_k\ \in\ {C}_j}}\frac{fs\left({g}_i,{p}_k\right)}{\left|{C}_j\right|} $$

That is, *β*(*g*_*i*_, *C*_*j*_) is the proportion of tissues in *C*_*j*_ that have *g*_*i*_ among their top alpha percent most-abundant proteins.

Let *score*(*S*,*p*_*k*_,*C*_*j*_) be the score of a protein complex *S* and a tissue *p*_*k*_ weighted based on the class *C*_*j*_. It is defined as:

$$ score\left(S,{p}_k,{C}_j\right)={\displaystyle \sum_{g_i\ \in\ S}} fs\left({g}_i,{p}_k\right)*\beta \left({g}_i,{C}_j\right) $$

The function *f*_*SNET*_(*S*, *X*, *Y*, *C*_*j*_) for some complex *S* is a t-statistic defined as:

$$ {f}_{SNET}\left(S,X,Y,{C}_j\right)=\frac{mean\left(S,X,{C}_j\right)- mean\left(S,Y,{C}_j\right)}{\sqrt{\frac{var\left(S,X,{C}_j\right)}{\left|X\right|}+\frac{var\left(S,Y,{C}_j\right)}{\left|Y\right|}}} $$

where *mean*(*S*,#,*C*_*j*_) and *var* (*S*,#,*C*_*j*_) are respectively the mean and variance of the list of scores {*score*(*S*,*pk*,*C*_*j*_) | *p*_*k*_ is a tissue in #}.

The complex *S* is considered differential (weighted based on *C*_*j*_) in *X* but not in *Y* if *f*_*SNET*_(*S*,*X*,*Y*,*C*_*j*_) is at the largest 5% extreme of the Student t-distribution, with degrees of freedom determined by the Welch-Satterwaite equation.

Given two classes *C*_*1*_ and *C*_*2*_, the set of significant protein complexes returned by SNET is the union of {*S* | *f*_*SNET*_(*S*,*C*_*1*_,*C*_*2*_,*C*_*1*_) is significant} and {*S* | *f*_*SNET*_(*S*,*C*_*2*_,*C*_*1*_,*C*_*2*_) is significant}; the former being complexes that are significantly consistently highly abundant in *C*_*1*_ but not *C*_*2*_, the latter being complexes that are significantly consistently highly abundant in *C*_*2*_ but not *C*_*1*_.

FSNET is identical to SNET, except in one regard:

For FSNET, the definition of the function *fs*(*g*_*i*_,*p*_*k*_) is replaced such that *fs*(*g*_*i*_,*p*_*k*_) is assigned a value between 1 and 0 as follows: *fs*(*g*_*i*_,*p*_*k*_) is assigned the value 1 if *g*_*i*_ is among the top alpha1% (default = 10%) of the most-abundant proteins in *p*_*k*_. It is assigned the value 0 if *g*_*i*_ is not among the top alpha2% (default = 20%) most-abundant proteins in *p*_*k*_. The range between alpha1% and alpha2% is divided into *n* equal-sized bins (default *n* = 4), and *fs*(*g*_*i*_,*p*_*k*_) is assigned the value 0.8, 0.6, 0.4, or 0.2 depending on which bin *g*_*i*_ falls into in *p*_*k*_. This tiered weighting system is termed fuzzification.

A test statistic *f*_*FSNET*_ is defined analogously to *f*_*SNET*_. Given two classes *C*_*1*_ and *C*_*2*_, the set of significant complexes returned by FSNET is the union of {*S* | *f*_*FSNET*_(*S*,*C*_*1*_,*C*_*2*_,*C*_*1*_) is significant} and {*S* | *f*_*FSNET*_(*S*,*C*_*2*_,*C*_*1*_,*C*_*2*_) is significant}.

For PFSNet, the same *fs*(*g*_*i*_,*p*_*k*_) function as in FSNet is used. But it defines a score *delta*(*S*,*p*_*k*_,*X*,*Y*) for a complex *S* and tissue *p*_*k*_ wrt classes *X* and *Y* as the difference of the score of *S* and tissue *p*_*k*_ weighted based on *X* from the score of *S* and tissue *p*_*k*_ weighted based on *Y*. More precisely: *delta*(*S*,*p*_*k*_,*X*,*Y*) = *score*(*S*,*p*_*k*_,*X*) – *score*(*S*,*p*_*k*_,*Y*).

If a complex *S* is irrelevant to the difference between classes *X* and *Y*, the value of *delta*(*S*,*p*_*k*_,*X*,*Y*) is expected to be around 0. So PFSNet defines the following one-sample t-statistic:

$$ {f}_{PFSNET}\left(S,\ X,\ Y,\ Z\right)=\frac{mean\left(S,X,Y,Z\right)}{se\left(S,X,Y,Z\right)} $$

where *mean*(*S*, *X*, *Y*, *Z*) and *se*(*S*, *X*, *Y*, *Z*) are respectively the mean and standard error of the list {*delta*(*S*,*p*_*k*_,*X*,*Y*) | *p*_*k*_ is a tissue in *Z*}. The complex *S* is considered significantly consistently highly abundant in *X* but not in *Y* if *f*_*PFSNet*_(*S*, *X*, *Y*, *X* ∪ *Y*) is at the largest 5% extreme of the Student t-distribution.

Given two classes *C*_*1*_ and *C*_*2*_, the set of significant complexes returned by PFSNet is the union of {*S* | *f*_*PFSNet*_(*S*,*C*_*1*_,*C*_*2*_,*C*_*1*_ ∪ *C*_*2*_) is significant} and {*S* | *f*_*PFSNet*_(*S*,*C*_*2*_,*C*_*1*_,*C*_*1*_ ∪ *C*_*2*_) is significant}; the former being complexes that are significantly consistently highly abundant in *C*_*1*_ but not *C*_*2*_, and vice versa.

### Fuzzy-FishNET

**Fig. 1:**
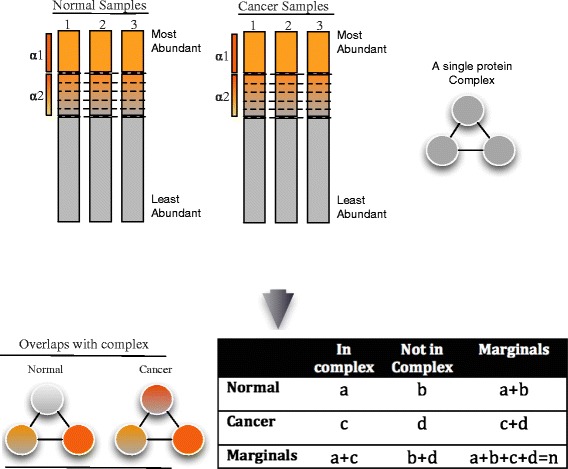
Schematic of Fuzzy-FishNET A vector of complexes are compared against protein weights (based on expression ranks). For each complex, a contingency table may be constructed based on the sum of weights between normal and cancer samples, and a *p*-value calculated based on the Fisher’s exact test

In Fuzzy-FishNET, a gene, gi, in sample pk, is assigned a weight ff(gi,pk) between 5 and 0 as follows: ff(gi,pk) is assigned the weight value 5 if gi is among the top alpha1% (default = 10%) of the most-abundant proteins in pk (Fig. 1). To boost sensitivity, a second alpha level, alpha2 is defined between the range top 10–20%. To account for the higher level of uncertainty for proteins in this region, weights are assigned based on ranks. To do this, proteins within alpha2 are divided into n equal-sized bins (default =4), and ff(gi,pk) and assigned a weight of 4, 3, 2, or 1 depending on which bin gi falls into in pk. Proteins that fall below the top 20% are assigned weights of 0.

For a complex S, and samples in class J, C_j_, and samples in class k, C_k_, the sum of weights can be expressed in a contingency table (Table 1) as shown below:

**Table 1:** A typical 2 x 2 contingency table

	In complex S	Not in Complex S	Marginals
Samples in Class J (C_j_)	a	b	a + b
Samples in Class K(C_k_)	c	d	c + d
Marginals	a + c	b + d	a + b + c + d = n

where a and c are the sum of weights for samples in class C_j_ and C_k_ mappable to proteins within complex S respectively, b and d are the sum of weights across samples in class C_j_ and C_k_ that are missed for proteins in complex S respectively. The Fisher exact probability p of obtaining this given set of values is then:

$$ p=\frac{\left(\begin{array}{c}\hfill a+b\hfill \\ {}\hfill a\hfill \end{array}\right)\left(\begin{array}{c}\hfill c+d\hfill \\ {}\hfill c\hfill \end{array}\right)}{\left(\begin{array}{c}\hfill n\hfill \\ {}\hfill a+c\hfill \end{array}\right)}=\frac{\left(a+b\right)!\left(c+d\right)!\left(a+c\right)!\left(b+d\right)!}{a!b!c!d!n!} $$

The Fisher exact probability *p* is also the hypergeometric probability of observing this particular arrangement of the data, assuming the given marginal totals, on the null hypothesis that both C_j_ and C_k_ have similar distributions of top alpha proteins across their class members mappable to constituent proteins belonging to complex S [37].

The *p*-value is calculated in a similar manner as in HE, as the sum of probabilities of obtaining an observation greater than or equal to *a*.

### Performance benchmarks (simulated data)

In simulated data, differential proteins are are defined *a priori* and used to construct pseudo-complexes at various levels of purity (i.e., the proportion of significant proteins in the complex).

Proteins in the same complex are expected to be expressionally correlated. To incorporate this principle in pseudo-complex generation, a Euclidean distance is calculated for all differential protein pairs across all samples. These are then clustered via Ward’s linkage. The differential proteins are reordered such that those with similar expression pattern are adjacent to each other. This reordered list is then split at regular intervals to generate 20, 101 and 62 differential pseudo-complexes for D1.2,D2.2 and RC1 respectively. An equal number of non-differential proteins are randomly selected, reordered based on expressional correlation, and then split to generate an equal number of non-differential pseudo-complexes.

The purity of the pseudo-complexes is lowered by decreasing the proportion of differential proteins [39]. This makes it harder for a differential pseudo-complex to be detected. So lowering purity tests for robustness and sensitivity. Here, purity is tested at three levels: 100%, 75% and 50%. At 100% purity, simulated complexes are comprised solely of differential proteins. These are randomly swoped out at lower purity levels; e.g. at 75% purity, 25% constituent differential proteins are randomly replaced with non-differential ones.

The differential and non-differential pseudo-complexes are combined into a single complex vector, which can be used for precision and recall-based evaluation of complex-based feature-selection methods:

$$ Precision = \frac{TP}{TP+FP}; Recall = \frac{TP}{TP+FN} $$

where TP, FP and FN are the True Positives, False Positives and False Negatives respectively. Since precision and recall are both important performance measures, they can be combined to generate an average. A common way of doing this is the F-score (*F*_*S*_) which is the harmonic mean between precision and recall:

$$ {F}_s=2*\frac{Precision\ *\  Recall}{Precision+ Recall} $$

### Performance benchmarks (real data)

On real data, differential complexes are not known *a priori,* so direct precision-recall analysis is not possible. Instead, one may test for reproducibility/stability [31, 34, 39].

Reproducibility can be gauged based on the overlaps between technical replicates. To compare the technical replicates in RC, let T_1_ and T_2_ be the significant complexes selected by independently applying a given network method on the two replicates. Then reproducibility may be measured as overlaps based on the Jaccard coefficient (J):

$$ J=\frac{\left|{T}_1{\displaystyle \cap }{T}_2\right|}{\left|{T}_1{\displaystyle \cup }{T}_2\right|} $$

This comparison may not be sufficiently robust and doesn’t allow evaluation at small sample sizes. So, one may perform resampling at different levels (resampling sizes of 4, 6 and 8) and generate a binary matrix for each method, where a value of 1 indicates significance at a significance level of 0.05 and 0 otherwise per complex. The binary matrix may be analyzed in 2 ways: Row summation to evaluate the numbers of predicted differential complexes and column summation to evaluate the stability of predicted differential complexes [34].

### Cross-validation (real data)

To demonstrate that Fuzzy-FishNET selects class-relevant differential complexes and only works when sample classes are real, cross-validation is performed 1000 times in two scenarios: where real classes exist(A) and where classes are shuffled/randomized (B) in RC. In each instance, half the data is used for feature-selection using Fuzzy-FishNET. A quarter of the remaining data is used for training, and the final quarter, validation. The classifier used is the deterministic Naïve Bayes method [40]. Cross-validation accuracy (CVAccuracy) is defined as:

$$ CVAccuracy=\frac{Number\  of\  correct\  class\  assignments}{Total\  size\  of\  validation\  set} $$

A good feature-selection approach will select features that can build highly accurate prediction models when true class labels are present. But if the class labels are shuffled, then this is expected to lead to strong decrease in predictive performance.

## Results and discussions

### F-score comparisons (Simulated datasets)

**Fig. 2:**
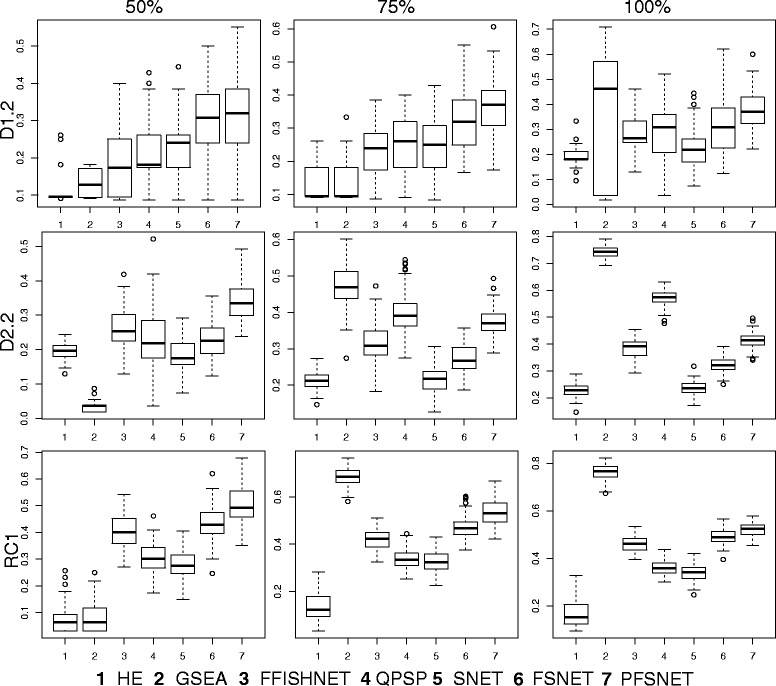
F-score distributions. The F-score distributions for several network-based methods are shown for three simulated datasets (D1.2, D2.2 and RC1) over three levels of purity (50, 75 and 100%)

The F-score distributions suggest that under noisier conditions (purity below 75%), Fuzzy-FishNET is an improvement over conventional HE and GSEA methods but overall appears to be a weaker method than earlier complex-based feature selection methods such as PFSNET (Fig. 2).

HE typically has the worst F-scores over all methods surveyed but in actuality, does very well in precision (Additional File 1) but falls short largely in recall (Additional File 2). Although GSEA is developed to address HE’s reliance on the unstable differential protein pre-selection step (e.g. based on the *t*-test), it is only powerful when purity is at 100%. If purity drops to 75% and below, GSEA’s F-score distributions quickly plummets. At purity of 50%, GSEA becomes the second worst method, beating only HE. Since noise and uncertainty in biological data is certainly expected, be it expressional or complexes, GSEA is unlikely a superior alternative to HE.

Fuzzy-FishNET’s performance is comparable to existing complex-based methods which also rely on the fuzzification process e.g. QPSP, FSNET, PFSNET. It does however, gain power as sample size increases. In D1.2, where n =3 (per class), Fuzzy-FishNET falls behind SNET even (the earliest incarnation, and least powerful of the RBNAs). But as sample size increases to n = 6 (per class) in RC1, then Fuzzy-FishNET beats most methods, is comparable to FSNET but weaker than PFSNET.

It is fascinating that swapping the differential protein selection step in favour of a fuzzy weighting system based on expressional ranks can greatly improve the precision-recall performance in HE. This is consistently observed over three sets of simulated data. However, there is no gold-standard for generating pseudo-complexes, nor is it known if the lack of biological coherence in the pseudo-complexes unfairly penalizes certain complex-based feature-selection approaches. Therefore it is also essential to consider results based on real data and real complexes for a comprehensive evaluation.

### Reproducibility of technical replicates (Real dataset)

Since technical replicates are present in RC. Each complex-based feature-selection method can be applied independently on each replicate. Inter-replicate overlaps is used as an indicator of complex-based feature-selection reproducibility.

Reproducibility is a strength of Fuzzy-FishNET (Table 2). Moreover, it does not make an overly large number of predictions, hence high-overlaps due to feature inflation or test hyper-sensitivity are not likely (misleading) contributors to its good performance.

**Table 2:** Selected features for Replicate 1 and 2 are shown alongside their intersections

	HE	GSEA	FFISHNET	QPSP	SNET	FSNET	PFSNET
Replicate 1	4	1	27	86	34	38	45
Replicate 2	6	2	28	75	32	39	46
Overlap	0.25	0.50	0.96	0.66	0.83	0.88	0.93

### Feature-selection stability (Real dataset)

Inter-replicate overlap is a good and simple way of evaluating reproducibility given that technical noise should be the only source of variability (and not due to biological/clinical heterogeneity). But resampling at various levels to evaluate feature-selection stability is also possible. This is useful, as it also allows explicit evaluation of feature-selection stability in the small sample-size scenario (most feature-selection methods do not work well when sample sizes are very small [41]).

Given the full RC dataset, random resamplings of sizes 4, 6 and 8 (representing small to moderate size sample size scenarios) followed by feature-selection are performed 1000 times. Two aspects are considered: the number of selected features at each resampling level, and the stability over all selected features.

**Fig. 3:**
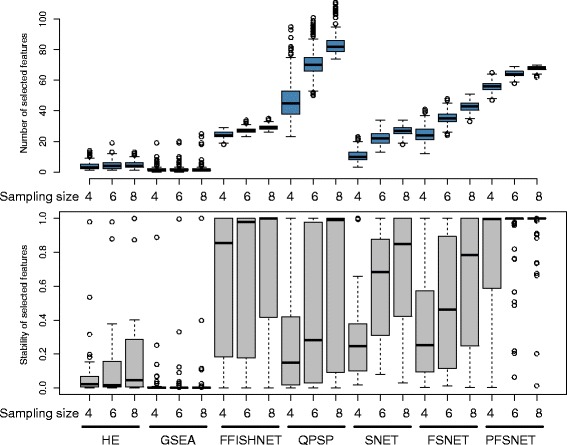
Differential complex-selection size and complex-selection stability (*Top*) Number of selected differential complexes across resampling sizes of 4, 6 and 8. (*Bottom*) Corresponding feature-selection stability of differential complexes at each resampling size

It is observed that HE, GSEA, Fuzzy-FishNET are particularly stable even as resampling size increases (Fig. 3 Top). While this is a good sign, it does not necessarily mean that the same features are selected each resampling round. Figure 3 (Bottom) shows that for HE and GSEA, feature-selection stability is particularly low. This is especially so for GSEA, possibly due to the presence of noise and uncertainty in real data. This supports the simulation observations.

On the other hand, Fuzzy-FishNET’s feature-selection stability is second only to that of PFSNET’s. However, PFSNET is more affected by sampling size increments, and it also selects considerably more features than Fuzzy-FishNET. There is a possibility that PFSNET may suffer from higher hyper-sensitivity and therefore feature-inflation issues than Fuzzy-FishNET.

### Comparing Fuzzy-FishNET with PFSNET

Both PFSNET and Fuzzy-FishNET do very well on reproducibility. In the previous section, as PFSNET selects more features and appears to be more affected by sampling size increments, it is possible it is relatively hyper-sensitive, thus leading to feature-selection inflation.

**Fig. 4:**
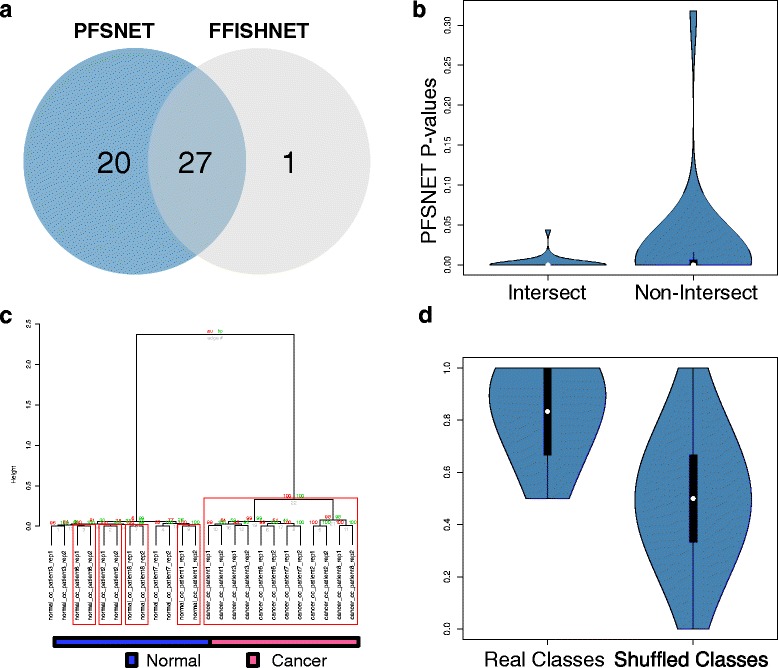
Differential complex agreement between Fuzzy-FishNET and PFSNET **a** Selected complex features in PFSNET and Fuzzy-FishNET (FFISHNET) overlap strongly. **b** Overlapping components are more significant given the *p*-values (**c**) The constituent proteins (found within Fuzzy-FishNET selected complexes) are highly discriminative between sample classes; red boxes signify highly stable branches based on bootstrap resampling (**d**) FFISHNET selected features are properly class-discriminative, i.e., provides a high accuracy when actual class differences exist

To determine if this is likely, significant features selected by PFSNET and Fuzzy-FishNET are compared (Fig. 4a), revealing deep overlaps (and therefore high agreements) between both methods. Since there are many more PFSNET complexes than Fuzzy-FishNET’s, the former’s *p*-values distributions for intersecting and non-intersecting complexes are compared, revealing that Fuzzy-FishNET selects higher quality complexes (Fig. 4b).

Unlike PFSNET, Fuzzy-FishNET doesn’t assign network scores for each complex per patient sample. However, class-discrimination analysis can still be performed using identified protein expressions found within significant complexes. Via hierarchical clustering (Ward’s linkage; Euclidean Distance) coupled to bootstrap resampling [42], the constituent proteins (found within Fuzzy-FishNET selected complexes) are highly discriminative between sample classes (Fig. 4c; red boxes signify highly stable branches within the tree structure).

Many Fuzzy-FishNET differential complexes are associated with ribosomal complexes [43], although some are also associated with the cytoskeleton [44], proteasome [45] and TNF-alpha complexes [46] (Additional File 3). Although these are consistent with previous observations [16], in the absence of actual experimental validation, it is better to withhold judgement based solely on expected functionalities with renal cancer.

Fuzzy-FishNET selected complexes that are also class-relevant, i.e., it doesn’t select features that are weakly associated with sample classes. The distribution of cross-validation (CV) accuracies when class labels are real, and when class labels are shuffled, reveals strong differences where the prediction model is far more accurate in the former than in the latter. If the feature-selection method selects a large number of irrelevant or weakly associated complexes (hyper-sensitive), then it is expected there will be little to no differences in CV accuracy between real and shuffled class labels.

### Determining the contribution of Rank Weights (Fuzzification) towards signal stability

The positive impact of incorporating fuzzification in Fuzzy-FishNET is not known. It is possible the method may work just as well with a uniform weight of 1 across the top alpha%. So, an unweighted version of Fuzzy-FishNET, FishNET is tested. Note that Fuzzy-FishNET is analogous to SNET’s uniform weight of 1 for the top alpha proteins, and 0 for all others.

**Fig. 5:**
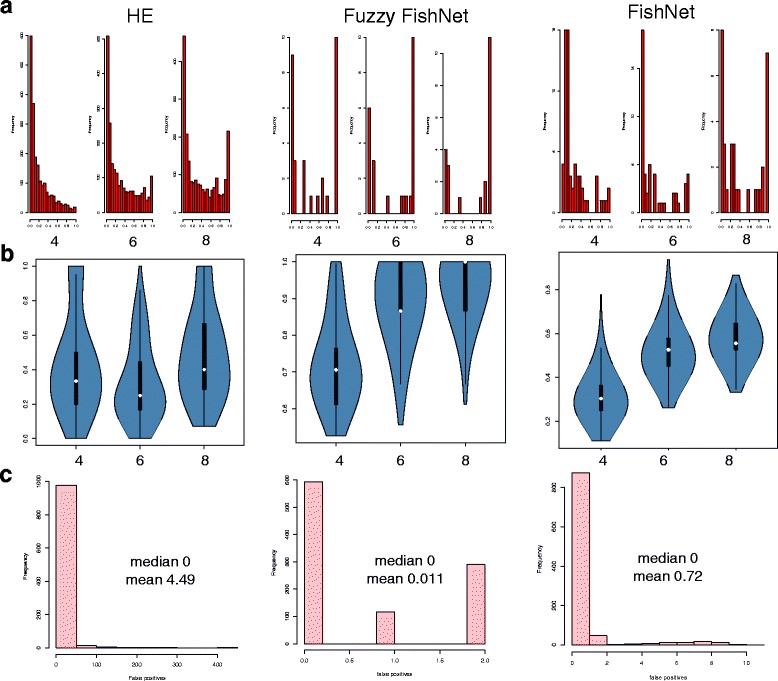
Comparing Fuzzy-FishNET with its non-fuzzy variant, FishNET and HE (**a**) Feature-selection stability for Fuzzy-FishNET and its non-weighted variant. HE is added on the left side for comparisons. **b** Pairwise feature-selection stability (**c**) False positive evaluations

Figure 5 shows the impact of fuzzification on Fuzzy-FishNET over three benchmarks: A/the frequency distribution of feature-selection stability, B/The pairwise feature-selection similarity based on the Jaccard distance and C/the frequency distribution of false positive rates based on random class assignment of RC’s normal samples into two pseudo-classes followed by feature-selection. Benchmarks A and B are shown over resamplings of sizes 4, 6 and 8. HE is included as a point of reference since it is a primordial version of Fuzzy-FishNET (and FishNET). FishNET is not a strong improvement over HE (given its weaker feature-selection stability), and thus it is clear that fuzzification has a very strong positive impact on feature-selection stability, as well as robustness against false positives. The most informative rank shifts lies within the most highly ranked proteins, and assigning higher weights to these, improves signal-to-noise ratios.

### Robustness towards alpha adjustment

As with the RBNAs, a valid concern is that alpha adjustments may lead to highly different differential complexes being selected. Parameterization of alphas however is not fixed: Increasing alpha from top 10% onwards can increase sensitivity, but comes at the cost of introducing more false positives as signal from lower ranked proteins are introduced into the complex scores (which is why lower weights for alpha2 are assigned). However, we would expect the top scoring complexes to be stable.

To examine this concern, the top ranked complexes generated from top alphas of 10, 20 and 30% is compared against the default Fuzzy-FishNET setting of alpha1 (top 10%) and alpha2 (top 10–20%) based on overlaps (A∩B/min(A,B).

The results are generally stable, with overlaps of 62% at alpha = 10, 64% at alpha = 20, and 69% at alpha = 30 respectively. However, alpha should not be set too low at the onset, as this will likely introduce many poorer quality complexes into the significant complex-based feature set too early into preliminary analysis.

## Conclusions

Fuzzy-FishNET is a powerful improvement over its predecessor, the hypergeometric enrichment (HE) approach. It differs only in the differential protein pre-selection step yet exhibits high precision-recall in simulated data while being the most reproducible over evaluations on real data. Based on cross-validations, it selects relevant features. Given these properties, Fuzzy-FishNET is a potentially powerful new entrant amongst complex-based feature-selection methods.

## Additional files

Additional file 1: Precision distributions for several network-based methods for three simulated datasets (D1.2, D2.2 and RC1) at three levels of purity (50, 75 and 100%). (PDF 98 kb)
Additional file 2: Recall distributions for several network-based methods for three simulated datasets (D1.2, D2.2 and RC1) at three levels of purity (50, 75 and 100%). (PDF 97 kb)
Additional file 3: Top 25 Fuzzy-FishNET complexes. (PDF 141 kb)
