# Supplementary material for: Fuzzy-FishNET: a highly reproducible protein complex-based approach for feature selection in comparative proteomics
Source: BMC Med Genomics. 2016 Dec 5;9(Suppl 3):67. doi: 10.1186/s12920-016-0228-z (PMC5260792; doi:10.1186/s12920-016-0228-z)

Additional File 1 Precision distributions for several network-based methods for three simulated datasets (D1.2, D2.2 and RC1) at three levels of purity (50, 75 and 100%).

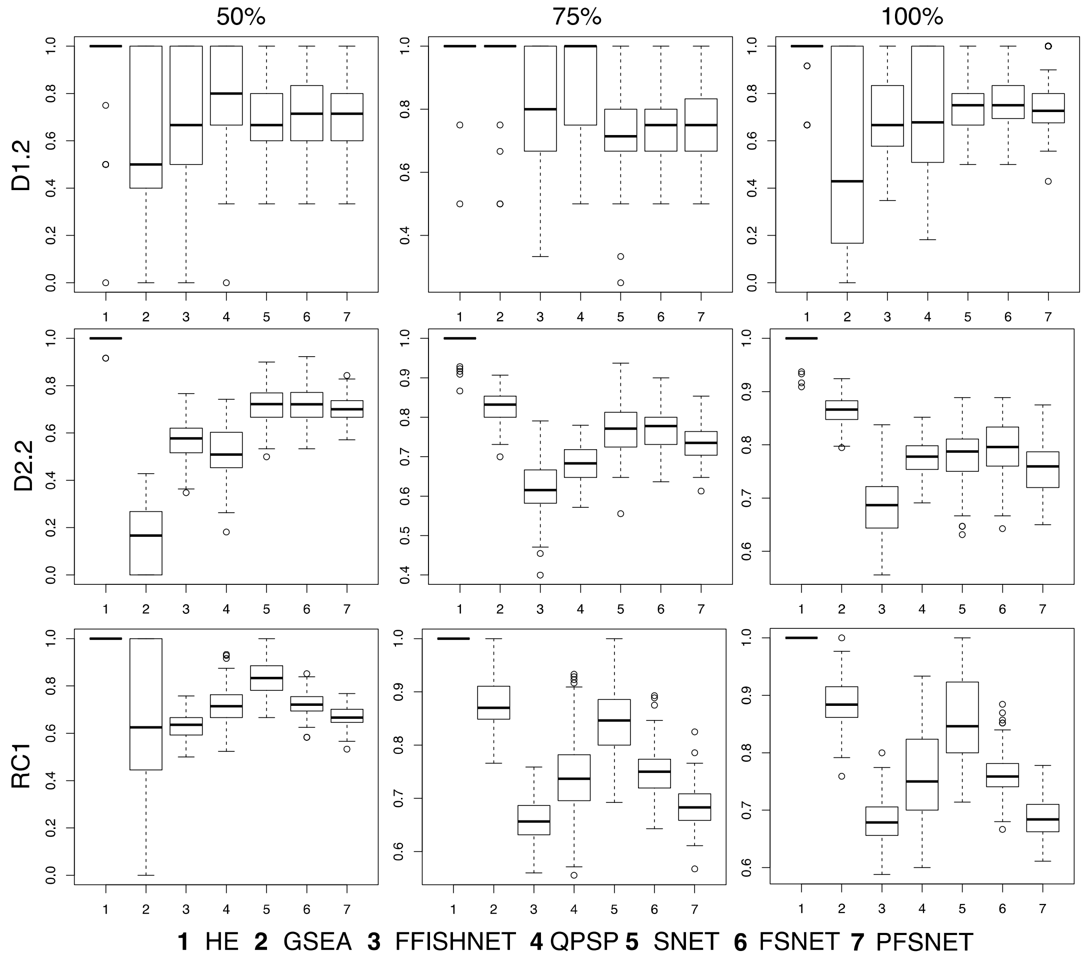

Supplement: Additional file 1: — Precision distributions for several network-based methods for three simulated datasets (D1.2, D2.2 and RC1) at three levels of purity (50, 75 and 100%). (PDF 98 kb) [file 12920_2016_228_MOESM1_ESM.pdf]
