# Supplementary material for: Fuzzy-FishNET: a highly reproducible protein complex-based approach for feature selection in comparative proteomics
Source: BMC Med Genomics. 2016 Dec 5;9(Suppl 3):67. doi: 10.1186/s12920-016-0228-z (PMC5260792; doi:10.1186/s12920-016-0228-z)

Additional File 2 Recall distributions for several network-based methods for three simulated datasets (D1.2, D2.2 and RC1) at three levels of purity (50, 75 and 100%).

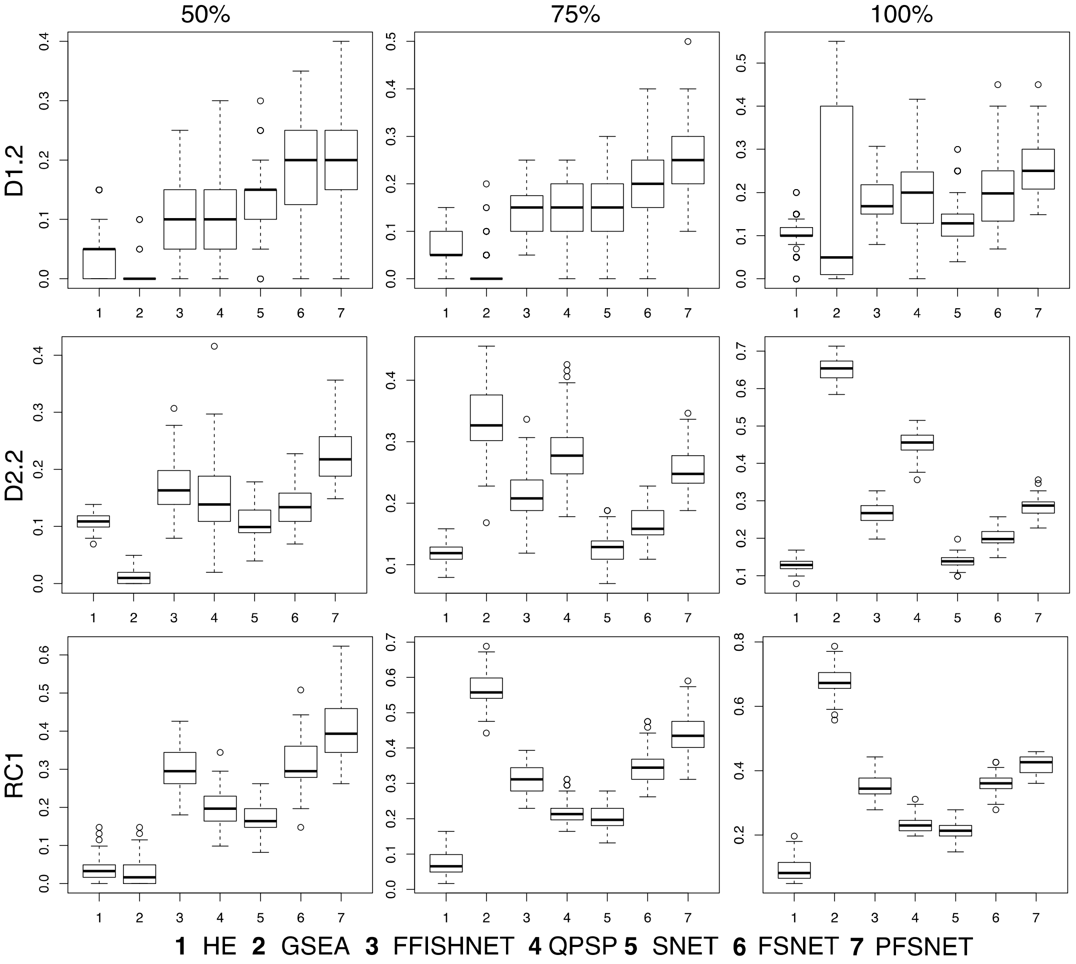

Supplement: Additional file 2: — Recall distributions for several network-based methods for three simulated datasets (D1.2, D2.2 and RC1) at three levels of purity (50, 75 and 100%). (PDF 97 kb) [file 12920_2016_228_MOESM2_ESM.pdf]
