# Supplementary material for: Fuzzy-FishNET: a highly reproducible protein complex-based approach for feature selection in comparative proteomics
Source: BMC Med Genomics. 2016 Dec 5;9(Suppl 3):67. doi: 10.1186/s12920-016-0228-z (PMC5260792; doi:10.1186/s12920-016-0228-z)
Supplement: Additional file 3: — Top 25 Fuzzy-FishNET complexes. (PDF 141 kb) [file 12920_2016_228_MOESM3_ESM.pdf]

Additional File 3 Top 25 Fuzzy-FishNET complexes

Complex ID	Complex Name	P-value	Functions
**306**	Ribosome, cytoplasmic	8.85E-48	protein biosynthesis
**3055**	Nop56p-associated pre-rRNA complex	1.06E-40	ribosome biogenesis
**308**	60S ribosomal subunit, cytoplasmic	5.88E-31	protein biosynthesis
**5380**	TRBP containing complex	4.86E-19	regulation of translation
**338**	40S ribosomal subunit, cytoplasmic	7.38E-19	protein biosynthesis
**1181**	C complex spliceosome	1.53E-14	rNA splicing
**351**	Spliceosome	2.93E-14	rNA splicing
**193**	PA700-20S-PA28 complex	6.15E-14	proteasomal ubiquitin-dependent protein catabolism
**181**	26S proteasome	6.69E-12	response to stress
**191**	20S proteasome	7.79E-11	response to stress
**194**	PA28gamma-20S proteasome	1.30E-10	response to stress
**5266**	TNF-alpha/NF-kappa B signaling complex 6	3.12E-10	I-kappaB kinase/NF-kappaB cascade
**1068**	12S U11 snRNP	8.60E-10	nuclear mRNA splicing, via spliceosome
**2174**	COP9 signalosome complex	1.12E-09	signal transduction
**192**	PA28-20S proteasome	1.52E-09	response to stress
**32**	PA700 complex	2.49E-09	proteasomal ubiquitin-dependent protein catabolism
**3040**	Multisynthetase complex	3.47E-08	tRNA ligase activity
**1737**	SF3b complex	2.94E-07	RNA splicing
**3082**	DGCR8 multiprotein complex	1.50E-06	RNA processing
**5613**	Emerin complex 25	3.39E-06	intracellular signaling cascade
**112**	Prefoldin complex	3.52E-06	cytoskeleton organization and biogenesis
**5232**	TNF-alpha/Nf-kappa B signaling complex	5.12E-06	I-kappaB kinase/NF-kappaB cascade
**1183**	CDC5L complex	5.26E-05	RNA splicing
**5199**	Kinase maturation complex 1	5.55E-05	protein kinase cascade
**27**	Arp2/3 protein complex	7.89E-05	actin cytoskeleton organization and biogenesis
